# The Quantitative Evaluation of the Density of the Segmental Branches of the MCA in Acute Ischemic Stroke Patients

**DOI:** 10.5041/RMMJ.10407

**Published:** 2020-10-14

**Authors:** Erdal Demirtaş, Ibrahim Oztoprak

**Affiliations:** 1 Department of Emergency Medicine, Sivas Cumhuriyet University Faculty of Medicine, Sivas, Turkey; 2 Department of Radiology, Sivas Cumhuriyet University Faculty of Medicine, Sivas, Turkey; 3 Department of Radiology, Canakkale Onsekiz Mart University Faculty of Medicine, Canakkale, Turkey

**Keywords:** Acute ischemic stroke, computed tomography, density, MCA Sylvian branches

## Abstract

**Aim:**

The aim of this study was to assess the density of the segmental branches of the middle cerebral artery (MCA) quantitatively as a predictor of acute ischemic stroke in patients without definitive infarct findings at cerebral parenchyma by non-contrast computed tomography (CT).

**Clinical rationale for the study:**

The clinical rationale for the study is to evaluate if the measurement of Sylvian fissure dot sign (SDS) would help early management of patients with stroke at the emergency department.

**Methods:**

Computed tomography scans of 101 patients admitted to the emergency department with stroke symptoms and/or signs were included in the study, retrospectively. In the patient group, the quantitative density of the segmental branches of the MCA in the Sylvian fissure was measured on the affected side and the contralateral side.

**Results:**

Quantitative density of SDS was significantly higher on the ischemic side of the brain. Receiver operating characteristic (ROC) analysis showed a cut-off value of 38.5 Hounsfield units (HU) as a predictor for acute ischemic stroke, with a sensitivity and specificity of 79% and 92%, respectively.

**Conclusion:**

Quantitative density of SDS on the affected side in patients without definitive cerebral infarct findings of parenchyma can be used in the emergency room as an objective predictor sign for the diagnosis of acute ischemic stroke. Considering this finding in the differential diagnosis of acute stroke patients in the emergency room has the potential to improve their clinical management, particularly for the patients without early parenchymal and vascular signs of stroke.

## INTRODUCTION

In stroke management every passing minute leads to significant catastrophic results until the diagnosis is made and treatment of the patient starts. For this reason, the diagnostic procedures should be performed appropriately to shorten this period.

Non-contrast cranial computer tomography (NCCT) is the first-line diagnostic test for emergency evaluation of acute stroke. Current approaches recommend detecting arterial occlusion with computed tomography (CT) or magnetic resonance (MR) angiography and selecting patients for endovascular treatment without delay. However, it is critical for clinicians to evaluate early signs of cerebral ischemia and interpret CT so that appropriate referrals can be made to centers where endovascular treatment or MR imaging can be performed.

The thrombus in the lumen of the Sylvian branches of the middle cerebral artery (MCA) is hyperdense relative to the normal vessel lumen and is seen as a dot within the hypodense fissure on axial CT sections among ischemic stroke patients. This finding on axial CT sections is called the Sylvian fissure dot sign (SDS).^1^

These signs were defined visually. Based on these data, quantitatively measuring the density of the SDS and determining a cut-off value could improve emergency service physicians’ ability both in detecting and interpreting early stroke signs and successfully managing the patients.

Recently, when we considered high patient expectation for early diagnosis of their illness at emergency departments, these diagnostic methods could improve emergency physicians’ skills. Herein, we aimed to analyze the density of SDS quantitatively and assign a cut-off density value for the patients with acute stroke symptoms and/or signs as a predictor. A standardized cut-off value could be helpful in detecting the dot sign objectively, particularly in patients without definite parenchymal or vascular signs of stroke.

In this paper, we aim to present the quantitative values of a finding that will help physicians, particularly those who work in secondary centers and decide to refer patients to a tertiary center dealing with anterior circulation brain infarcts.

## MATERIALS AND METHODS

### Study Population

This retrospective study was approved by the institutional human ethics committee (2019-02/39). The medical charts (clinical database of emergency department and hospital patient medical records) were evaluated to reveal the patients managed for acute ischemic stroke, according to the ICD codes (I60, I62, I62, I63, I64, I65, I66, I67) from January 2012 to December 2018 at our tertiary university hospital. Patients were included if they had NCCT imaging on admission in the emergency department and repeated 24 hours later after admission during their stay in the neurology department.

We identified 1,283 patients diagnosed with acute cerebrovascular stroke admitted to the emergency department and hospitalized at neurology and/or intensive care units. We included those patients in the study who fulfilled our inclusion criteria: infarcts involving the anterior circulation of the brain; CT examination within 6 hours from the onset of symptoms; no evidence of other pathologies or cerebral hemorrhage on CT images; and no findings of cerebral parenchymal infarct on CT images. Exclusion criteria were as follows: traumatic brain injury, encephalitis, meningitis, hemorrhagic stroke, previous history of any neurosurgery, and infarcts in the posterior circulation of the brain.

After applying the inclusion/exclusion criteria, 101 patients with NCCT scans were enrolled in the study. Data regarding age, gender, and CT were collected from medical charts, and results from complete blood cell count, kidney and liver function tests, and bleeding parameters were noted.

### Control Group

The control group consisted of the patients with normal NCCT presenting to the emergency department with a complaint of headache without any diagnosis of vascular or rheumatologic disease. Age and sex distribution were similar in both patient and control groups. The artery density within the M2 and M3 branches of the MCA in the Sylvian fissure was measured.

### Evaluation of Non-contrast Cranial CT Imaging

All CT examinations were acquired with a 128-slice CT scanner (Aquilion, Toshiba Medical Systems, Tokyo, Japan). Axial images with 2-mm slice thickness were obtained. All CT scans were analyzed by a neuroradiologist with 20 years of experience in this field.

A 1-mm^2^ region of interest (ROI) was used to measure the SDS density in Hounsfield units (HU). At least three measurements were carried out in each patient, and the maximum SDS density was recorded.

Neuroradiology evaluation comprised two steps. Firstly, parenchymal signs of acute ischemia were searched for. These signs include: hypo-attenuating brain parenchyma compared to the symmetric area on the contralateral side, loss of gray–white matter differentiation, insular ribbon sign, effacement of basal ganglia, obscuration of lentiform nucleus, and sulcal effacement. Patients without any of these signs at baseline NCCT, and developing signs of cerebral ischemia at control NCCT, were included in the study.

**Figure 1:**
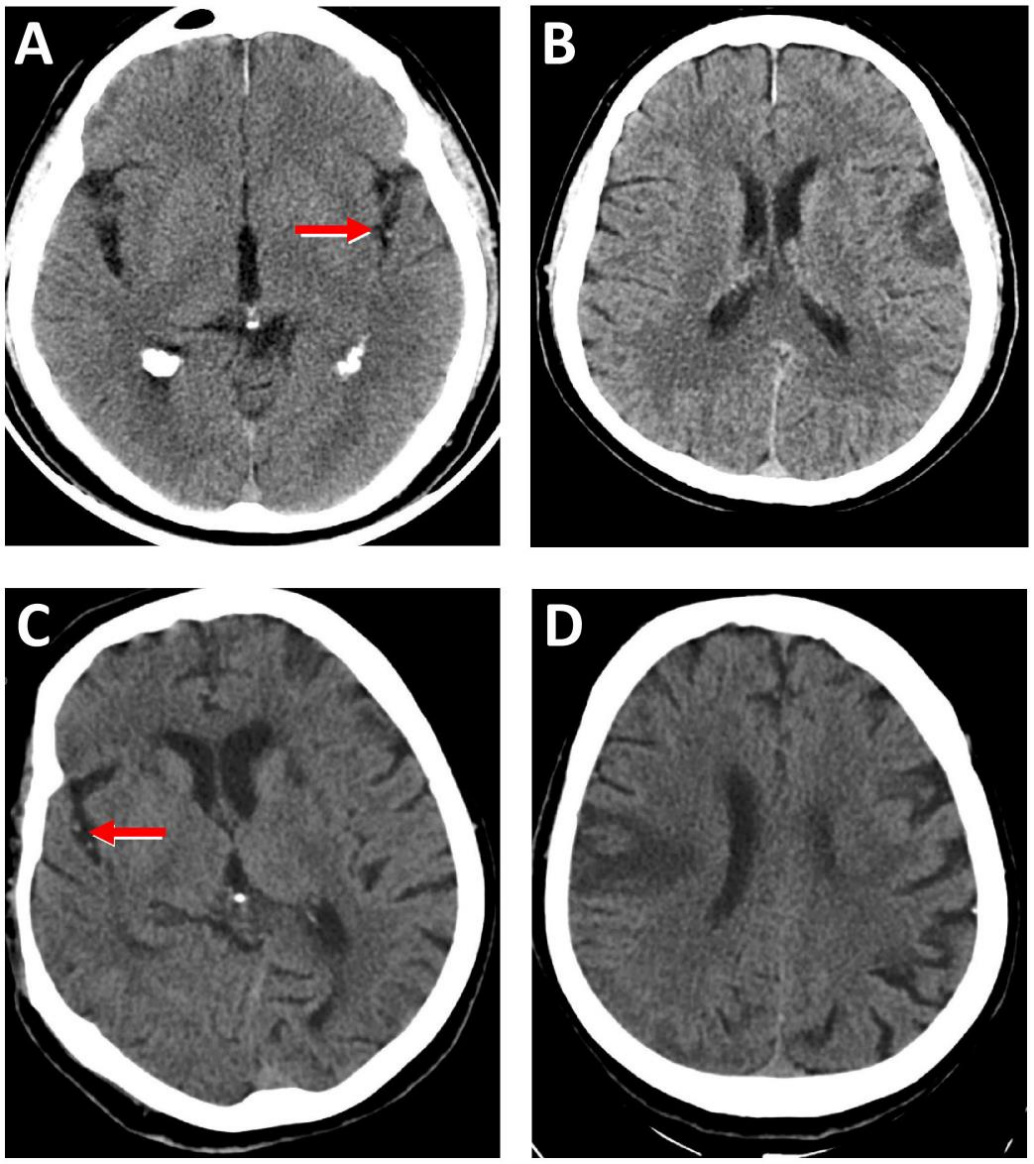
Increased Density of MCA Branch. NCCT scan of head demonstrates the increased density of the branch of MCA in the Sylvian fissure (red arrow) at admission of acute ischemic cerebral stroke to the emergency department (**A, C**). Confirmation of cerebral ischemia 24 hours later after admission (**B, D**).

Secondly, visual evaluation was made of the M2 and M3 branches of the MCA. The density of the dot sign was measured in patients with visually detected high attenuation when compared with the contralateral side. The density measured in the dot sign was defined as the value of quantitative dot sign. The SDS density was measured at the M2 and M3 segments of MCA in Sylvian fissure. In the control group, SDS density was measured at the M2 and M3 segments of MCA in Sylvian fissure bilaterally. Figure 1 shows an obvious dot sign on CT images acquired at the time of the admission to the emergency department (Figure 1, Panels A and C). The control CT images obtained 24 hours after admission clearly show parenchymal ischemic areas (Figure 1, Panels B and D).

### Statistical Analysis

All statistical analyses were performed using IBM SPSS Statistics for Windows version 23.0 (IBM, Armonk, NY, USA), and continuous data were reported as mean±standard deviation (SD) or median (minimum–maximum); categorical data were given as number and percentage. Kolmogorov–Smirnov test was used as a test for normality for continuous data, and parametric paired and unpaired *t* tests were used. Receiver operating characteristic (ROC) analysis was used to determine the diagnostic efficiency of quantitative SDS. The correlation analyses were done by Spearman correlation test. The correlation coefficients were interpreted as very strong (*r*≥0.80), strong (0.79≥*r*≥0.60), moderate (0.59≥*r*≥ 0.40), weak (0.39*≥*r≥0.20), and very weak (0.19≥*r*); *P*<0.05 was considered statistically significant.

## RESULTS

In the patient group, 49% of the patients were male (*n*=50), and 51% (*n*=51) of the patients were female. Mean age of the patient group was 71.4 years, with a range of 22–96 years.

In the control group, 39% of the patients were male (*n*=40), and 61% (*n*=61) of the patients were female. Mean age of the patient group was 70.3 years, with a range of 24–86 years.

**Figure 2:**
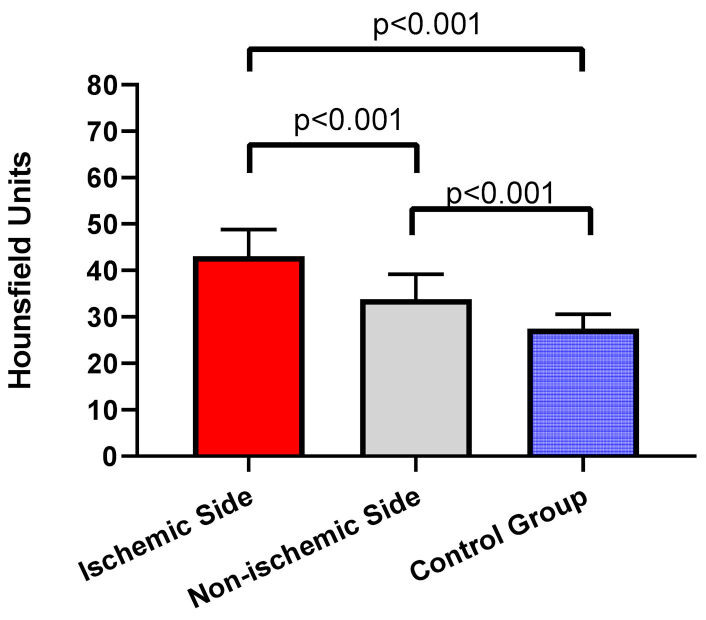
Comparisons of SDS Density. In the patient, the value of SDS density on the ischemic side was significantly higher than that on the non-ischemic side and the control group (*P*<0.001). The SDS density on the non-ischemic side was significantly higher than the SDS density of the control group (*P*<0.001).

Figure 2 shows the comparison of the quantitative density of SDS on the ischemic side of the patient and that of the non-ischemic side of the patient and the control group. In acute ischemic stroke patients, the quantitative density of SDS was significantly higher than that on the non-ischemic side (43.1±5.8 versus 33.8±5.4; *P*<0.001). Also, SDS density was significantly higher on the affected side when compared with that of the control patients (43.1±5.8 versus 30.1±4.2; *P*<0.001).

The SDS density on the non-ischemic side of the patients was also significantly higher than that of the control patients (33.8±5.4 versus 30.1±4.2; *P*< 0.001).

As shown in Table 1, a positive mild correlation was found between the density of dot sign and hematocrit, hemoglobin, and red blood cells (RBC) (*r*=0.210, 0.235, 0.305, respectively; *P*<0.005), and a negative mild correlation between the density of SDS and international normalized ratio (INR) (*r*= −245; *P*<0.05).

**Table 1:** Correlations between Blood Parameters and Dot Sign Density.

Parameters	Dot Sign HU	
	*r* value	*P* value
Hematocrit, %	0.210	0.017
Hemoglobin, g/dL	0.235	0.009
INR	−0.248	0.010
RBC	0.305	0.001

HU, Hounsfield unit; INR, international normalized ratio; RBC, red blood cell count.

**Figure 3:**
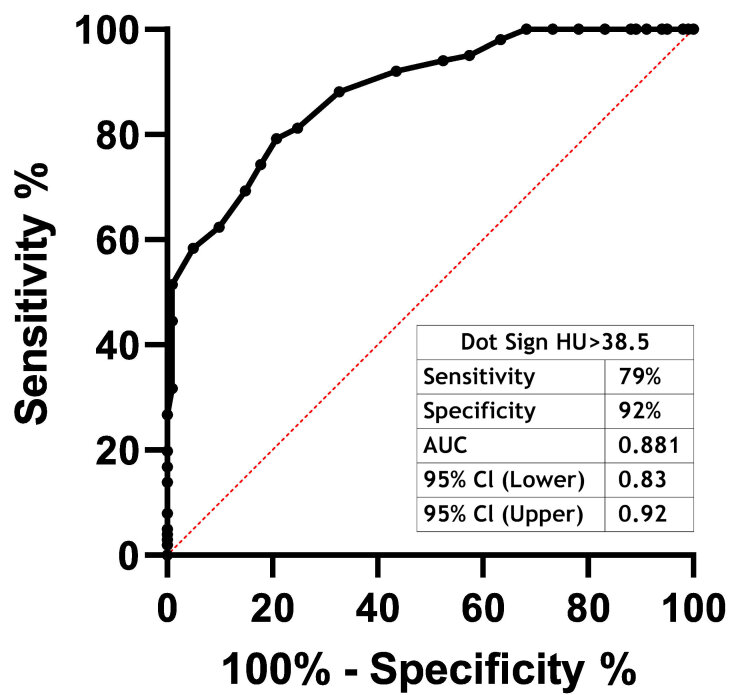
The Receiver Operating Characteristic Curve of SDS Density in Patients with Acute Ischemic Cerebral Stroke. The receiver operating characteristic curve of the density of SDS in patients with acute ischemic cerebral stroke has discriminative ability, with an area under the curve (AUC) of 0.881 (sensitivity 79%, specificity 92%).

In Figure 3 ROC analyses demonstrate the performance of the density of SDS in the prediction of acute ischemic stroke. We found that the density of SDS had a significant discriminative ability, with an area under the curve (AUC) of 0.881, a sensitivity of 79%, and specificity of 92% (*P*<0.001).

## DISCUSSION

In acute ischemic stroke, multiple neuronal deaths occur every minute. Clinicians race against time in the suspected diagnosis of stroke. If treatment can be started immediately, it may also be possible to salvage ischemic tissue. Our study showed that thrombosed MCA Sylvian branches can be quantitatively detected by using density measurement when the thrombus is not detected on visual evaluation of non-contrast CT imaging.

Dot sign density has been measured for many purposes in the literature. Barber et al. showed that dot sign was seen more frequently than hyperdensity of the middle cerebral artery (HMCA) and it was related with better prognosis.^2^ Li et al. conducted a study to compare the neurological recovery after intravenous recombinant tissue-type plasminogen activator (rtPA) in patients with or without proximal hyperdense middle cerebral artery sign (p-HMCAS) and in patients with or without the distal hyper-dense middle cerebral artery sign (d-HMCAS). They noted that patients with p-HMCAS were less likely to have rapid neurological recovery than those with d-HMCAS. Also, patients with p-HMCAS were associated with more severe neurological deficit and less rapid neurological recovery than patients with d-HMCAS.^3^

The SDS can be considered as a vascular sign. Leary et al.^4^ compared the diagnostic performance of MCA dot sign on NCCT with conventional cerebral angiography. They detected the dot sign in 16.5% of the patients. They reported 38% sensitivity and 100% specificity of the dot sign in detecting thrombus in M2 and M3 segments of MCA. They proposed the dot sign as an early helpful finding in detecting acute cerebral ischemia. They evaluated the density in the SDS visually.^4^ However, in this study we measured it quantitatively. This resulting cut-off value could improve the detection of dot sign before it becomes visually detectable.

The results of the studies mentioned above are concordant with the present study results. The SDS is an early sign of an acute ischemic event. Therefore, detecting the SDS is important for a physician. Quantifying the density of SDS could help physicians to diagnose the patient in the early phase. Our results suggest that quantifying the density of SDS could improve the sensitivity and specificity of the identification of acute ischemic stroke.

In the literature, we found only few studies that quantitatively measure the density of SDS. Previously reported studies showed that results differed among raters when only visual analysis of the early signs of acute stroke was carried out.^5^ Regardless of the physician’s personal skills, the standardization of measurements (including a cut-off density value) for SDS could be helpful in the early detection of acute stroke.

Our results showed a 38.5-HU cut-off value in the M2 and M3 segments of MCA for detecting ischemic stroke. Previously, a combination of visual assessment and additional attenuation measurement with a cut-off value of 42.5 HU was recommended for the most sensitive and reliable detection of MCA occlusion on the NCCT.^6^ We measured MCA density in more distal branches, and this might explain the different cut-off value in our study compared to that of other studies.

In their study, Topcuoglu et al. measured the density of hyperdense MCA and dot sign of the patients presenting with the signs of acute cerebral ischemia by NCCT, before and after the treatment. They found a mean SDS density of 44.5±5.7 HU on the ischemic side. At the same time, they suggested that hyperdense artery findings may have a prognostic and diagnostic use in cases where CT angiography cannot be performed; however, instead of qualitative evaluation, a quantitative assessment would be more useful to clinical correlation.^7^

The diagnostic value of the density of HMCA in ischemic infarcts has been analyzed by Abd Elkhalek and Elia.^8^ They found a HMCA density in the range of 44–58 HU (mean 47 HU). In that study, comparing the density of MCA on the affected and non-affected sides showed 100% sensitivity and 60% specificity. In our study, we found a density of SDS 43.1±5.8 HU on the ischemic side, similar to the study by Abd Elkhalek and Elia.^8^ We think that the cut-off density values obtained in our study could be used in the emergency department in patients with suspected acute cerebral ischemia.

It should be kept in mind that the blood density in the arteries will not be the same in all cases and that it may be dependent on different parameters. Black et al. measured the density in venous sinuses and analyzed the correlation of it with hemoglobin and hematocrit values. They reported a positive correlation between these parameters.^9^ In our study, we found a positive correlation between the measured density of SDS and hemoglobin and hematocrit that was similar to the studies mentioned above. In addition, we found that RBC has a positive and INR has a negative correlation with density value. This result suggested that particularly iron-containing proteins in the vessel increased the density. Although these correlations were weak, they could affect the density measurements. Therefore, the clinician should be aware of the need to take some laboratory tests into account.

In the previous studies, dot sign density was measured for other purposes. The findings from this study suggest that a quantitative measurement of SDS in patients with suspected acute cerebral ischemia could be used as a rapid predictor sign. In this context, we think that the emergency physician will make a more accurate decision by measuring the density quantitatively rather than making a visual assessment. This is the main clinical implications of our study.

The main limitations of this study were its retrospective design and the enrollment of patients from a single center. All measurements were done by one physician. Thus inter-rater variability was not assessed. We hope that, in further studies with patient groups with or without comorbidities or cardiovascular drug use, the feasibility of quantitative measurement of SDS can be investigated for use in the emergency department.

In conclusion, during the assessment of brain NCCT in the emergency department, the quantitative measurement and comparison of the density of SDS could be very helpful particularly for the patients without definitive infarct findings of cerebral parenchyma.

## CLINICAL IMPLICATION/FUTURE DIRECTIONS

In particular, dot sign density measurement can be used as a complementary tool in the evaluation of the CT of patients suspected of acute stroke in the second step emergency services, so that appropriate referrals can be made for thrombolytic or interventional procedures.

There is a need to increase the early recognition, in the emergency department, of cerebral infarct and thus reduce the burden of acute ischemic stroke on our health system.

## Abbreviations

AUC: area under curve
CT: computed tomography
d-HMCAS: distal hyperdense middle cerebral artery sign
HMCA: hyperdensity of the middle cerebral artery
HU: Hounsfield unit
MCA: middle cerebral artery
NCCT: non-contrast cranial computed tomography
p-HMCAS: proximal hyperdense middle cerebral artery sign
RBC: red blood cells
ROC: receiver operating characteristic
rtPA: recombinant tissue-type plasminogen activator
SD: standard deviation
SDS: Sylvian fissure dot sign

## References

[1] Shetty SK (2006). The MCA dot sign. Radiology.

[2] Barber PA, Demchuk AM, Hudon ME, Pexman JH, Hill MD, Buchan AM (2001). Hyperdense sylvian fissure MCA “dot” sign: a CT marker of acute ischemia. Stroke.

[3] Li Q, Davis S, Mitchell P, Dowling R, Yan B (2014). Proximal hyperdense middle cerebral artery sign predicts poor response to thrombolysis. PLoS One.

[4] Leary MC, Kidwell CS, Villablanca JP (2003). Validation of computed tomographic middle cerebral artery “dot” sign: an angiographic correlation study. Stroke.

[5] Wardlaw JM, Farrall AJ, Perry D (2007). Factors influencing the detection of early CT signs of cerebral ischemia: an internet-based, international multiobserver study. Stroke.

[6] Ernst M, Romero JM, Buhk JH, Kemmling A, Fiehler J, Groth M (2014). Sensitivity of visual and quantitative detection of middle cerebral artery occlusion on non-contrast-enhanced computed tomography. Neuroradiology.

[7] Topcuoglu MA, Arsava EM, Akpinar E (2015). Clot characteristics on computed tomography and response to thrombolysis in acute middle cerebral artery stroke. J Stroke Cerebrovasc Dis.

[8] Abd Elkhalek YI, Elia RZ (2016). Qualitative and quantitative value of hyperdense MCA sign as a prognostic marker for infarction. The Egyptian Journal of Radiology and Nuclear Medicine.

[9] Black DF, Rad AE, Gray LA, Campeau NG, Kallmes DF (2011). Cerebral venous sinus density on noncontrast CT correlates with hematocrit. AJNR Am J Neuroradiol.

